# Assessment of Factories on Adherence to COVID-19 Standard Operating Procedures: A Case Study of Wakiso, Mukono, Buikwe, and Jinja Districts, Uganda

**DOI:** 10.1155/2024/6670510

**Published:** 2024-01-16

**Authors:** Joseph M. Kungu, Edity Namyalo, Angella Musewa, Sarah Nitumusiima, Phiona Keije, Catherine Nakakooza, Osborn Oyirwoth

**Affiliations:** ^1^College of Veterinary Medicine Animal Resources and Biosecurity, Makerere University, Kampala, Uganda; ^2^Africa One Health Universities Network, Kampala, Uganda

## Abstract

**Background:**

Coronavirus disease (COVID-19) is an infectious disease caused by the SARS-CoV-2 virus. Uganda confirmed the first case of COVID-19 on 21st March, 2020, which led to the first total lockdown in the country. During the lockdown, some factories remained operational; hence, there is a need for a study aimed at assessing the level of adherence to COVID-19 standard operating procedures (SOPs) in factories as a mitigator for the pandemic.

**Methods:**

A cross-sectional study to assess compliance of factories to COVID-19 SOPs was conducted in Wakiso, Mukono, Buikwe, and Jinja districts during the month of September, 2021. This involved visitation of factories and collection of data using the KoboCollect tool by interviewing general managers as well as human resource managers of the factories. A total of 39 factories were included in the study and were categorized into four major groups; food and beverages (15), plastics (5), construction (8), and others (11). Data analysis was done using STATA version 14.2.

**Results:**

Overall adherence to COVID-19 SOPs by the factories was 64.1% (95% CI = 49.1–79.1). Communication and training of employees (79.5%; 95% CI = 66.8–92.2), wearing personal protective equipment (PPE), and respiratory protection (79.5%; 95% CI = 66.8–92.2) as well as enhanced cleaning and disinfection of surfaces (74.4%; 95% CI = 60.6–88.1) were the most implemented SOPs in the factories. Implementation of the SOPs was the highest in Mukono district (88.9%; 95% CI = 68.4–100) and the least in Wakiso district (40.0%; 95% CI = 9.6–70.4). The COVID-19 SOPs were followed mostly in construction material factories (87.5%; 95% CI = 64.6–100) and least in food and beverage factories (40%; 95% CI = 15.2–64.8). There was no significant difference in the adherence of COVID-19 SOPs among the districts (*X*^2^ = 5.02 and *P*=0.17) and factories (*X*^2^ = 7.04 and *P*=0.07). Although good overall adherence to SOPs noted was not dependent on location and type of factory, adherence to some SOPs such as exposure control plan, presence of signages on COVID-19, maintenance of social distance, and implementation of a health control plan varied with location of the district. Likewise, exposure control plan, signages on COVID-19, and maintenance of social distance varied significantly with the type of factory.

**Conclusion:**

This study revealed moderately good overall adherence to COVID-19 SOPs by factories, with variations in the level of implementation of individual SOPs being observed.

## 1. Background

Coronavirus disease (COVID-19) is caused by the SARS-CoV-2 virus, which is characterized with a wide range of symptoms such as sore throat, fevers, headaches, congestion, or runny nose among others and it affects all age groups. It is transmitted when people inhale air with contaminated droplets and through contact with contaminated surfaces [1].

Globally, the cumulative number of cases was 219,000,000 million cases, including 4,550,000 million deaths as of 16^th^ September 2021 [1]. Uganda confirmed the first case of COVID-19 on 21^st^ March, 2020 [2] leading to the first total lockdown in the country. As of 16^th^ September 2021, a total of 122,000 cases and 3,103 deaths had been reported across all districts in the country [3]. Since the last Presidential address on COVID-19 on 29^th^ May 2021, the COVID-19 situation drastically changed. On 4^th^ June 2021, the country registered the highest number of cases in a single day, 1,259 cases out of 7,424 tests done (17% positivity rate), reflecting an upsurge in case patients since the end of March 2021, following a 3-month period (January, February, and March 2021) of controlling the epidemic [2, 4]. Uganda registered an exponential increase in the number of confirmed cases that cut across all the sectors including the industrial sector.

During the first lockdown, Uganda's exports declined from US$383.62 million in January 2020 to US$352.91 million in February 2020 and imports declined from US$711.99 million in January 2020 to 593.79 million in March 2020 [4, 5]. By 22nd April, 2020, 4200 factories had been closed because of the pandemic, leaving only 215 in operation on the condition that very strict SOPs would be followed [4].

Uganda Manufacturers Association was engaged by the Ministry of Health to ensure continuity of essential commodity production by factories. The meeting aimed at providing and emphasizing the Ministry's guidelines to reduce the risk of contracting and spreading the virus among the factory workers and the public [2].

Several Nonpharmaceutical Interventions (NPIs) have been reported to play a vital role in preventing as well as slowing down of communicable disease outbreaks [6, 7]. It was upon this note that a number of NPIs constituted the guidelines or SOPs set up to manage the outbreak of the novel COVID-19 pandemic globally [8]. Previous studies reported low adherence to COVID-19 SOPs of 29% and 12.3% in Uganda and Ethiopia, respectively [9, 10]. Another study conducted among supermarkets in Uganda revealed that only 16.6% of the supermarkets complied with the COVID-19 prevention and control guidelines [11].

Some studies reported frequent hand washing as the most practiced preventive measure [9, 10, 12], while others recorded wearing of masks and social distancing as the key SOPs [13–15].

Factory workers spend most of their time at work, with a high tendency of interaction through sharing of work space and utilities, putting them at high risk of COVID-19 infection [4, 12]. Thus, the study aimed at determining the level of adherence and compliance of the selected factories to the recommended SOPs. These findings aimed to identify areas for improvement during planning for pandemic preparedness, readiness, and response to future outbreaks.

## 2. Methods

### 2.1. Study Design

A cross-sectional study to assess compliance of factories to COVID-19 SOPs was conducted in the districts of Wakiso, Mukono, Buikwe, and Jinja in September, 2021. These four districts host the highest number of factories in Uganda.

**Figure 1:**
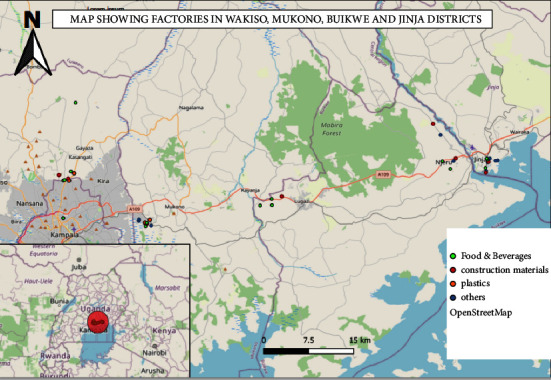
A map showing the distribution of factories in the four districts.

All operational factories in the study districts were mapped by a team (Team Leader from Ministry of Health, District Surveillance Focal Person (DSFP), AFROHUN Fellow) with guidance from Uganda Manufactures Association (UMA), Private Sector Foundation (PSF), and the District Local Governments (Figure 1). The DSFP together with the team compiled a list of factories in each district (Wakiso district had 53 factories, Buikwe had 50 factories, Mukono had 58 factories, and Jinja had 59 factories) based on the population of workers, activities, engagement of workers, and registration status of the factory. A maximum of 10 factories were then selected by simple random sampling per district of study (Table 1).

### 2.2. Data Collection

Data were collected using a digitalized tool designed and tested in KoboCollect software application and based on the checklist assessing evidence of NPIs developed by the US-National Institute of Health [16].

### 2.3. Data Analysis

The data were exported from KoboCollect as a Microsoft Excel file and cleaned. They were then analyzed using STATA 14.2 to generate frequencies and percentages, and the Kruskal–Wallis test was conducted to determine if there was a relationship between adherence to COVID-19 SOPs and type of factories and location of districts.

## 3. Results

A total of 39 factories were surveyed from Buikwe [10], Jinja [10], Wakiso [10], and Mukono [9]. Factories sampled were categorized into four major groups, namely, food and beverages (15/39), plastics (5/39), construction (8/39), and others (11/39). There was no significant relationship between the district location and type of factory (*X*^2^, *P*=0.08), as shown in Table 2.

**Figure 2:**
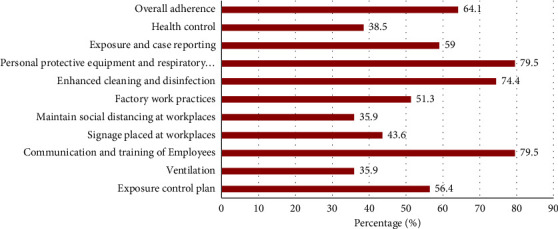
Frequency of factories adherence to COVID-19 SOPs.

The overall adherence to the SOPs was observed to be good in 64.1% (95% CI = 49.1–79.1) among the factories assessed. It was observed that the most implemented SOPs (79.5%; 95% CI = 66.8–92.2) were communication and training of employees, wearing of personal protective equipment (PPE), and respiratory protection. This was followed by enhanced cleaning and disinfection of surfaces (74.4%; 95% CI = 60.6–88.1). Ventilation (35.9%; 95% CI = 20.8–51.0), social distancing (35.9%; 95% CI = 20.8–51.0), display of COVID-19-related signages (43.6%; 95% CI = 28.0–59.1), and health control plans (38.5%; 95% CI = 23.2–53.7) were poorly implemented in the factories, respectively, as shown in Figure 2.

Out of 10 SOP attributes assessed, only exposure control plan, presence of signages on COVID-19, maintenance of social distance, and implementation of a health control plan were observed to have a significant relationship with location of district.

Implementation of an exposure health control plan was practiced the highest in factories of Mukono district 9/9 (100.0%) and least in Jinja district 1/10 (10.0%; 95% CI = 8.6–28.6) and Wakiso 1/10 (10.0%; 95% CI = 8.6–28.6). Maintenance of social distance was followed the most in Jinja district 8/10 (80.0%; 95%CI = 55.2-100) and Mukono district 6/9 (66.7%; 95% CI = 29.6–90.4) factories and lowest in both Buikwe (0.0%) and Wakiso (0.0%). Factories in Buikwe district highly displayed COVID-19-related signages at workplaces 7/10 (70.0%; 95% CI = 41.6–98.4) unlike in Mukono district 1/9 (11.1%; 95% CI = 9.4–31.6). All factories in Mukono district had health control measures in place unlike in Jinja and Wakiso districts where only one factory had these measures (10%; 95% CI = 8.6–28.6) (Table 3). Good overall adherence to SOPs was observed to be the highest in Mukono district (88.9%; 95% CI = 68.4–100), followed by Buikwe (70.0%; 95% CI = 41.6–98.4) and Jinja (60%; 95% CI = 29.6–90.4) and least in Wakiso district (40.0%; 95% CI = 9.6–70.4) as shown in Table 3.

Only maintenance of social distance (*X*^2^ = 11.24, *P* = 0.03) as well as exposure and case reporting (*X*^2^ = 11.24, *P* = 0.01) were the SOP attributes observed to have a significant relationship with the type of factory assessed. Social distancing was well maintained in other types of industries (72.7%; 95% CI = 46.4–99.0) and poorly observed in construction and plastics as well as food and beverages factories. Exposure and case reporting was implemented highly in construction factories (87.5%; 955% CI = 64.6–100), followed by the category of other unspecified factories (81.8%; 95% CI = 59.0–100) and plastics (60.0%; 95% CI = 17.1–100) and least in food and beverages (26.7%; 95% CI = 4.3–49.0).

It was observed that construction material factories generally had good overall adherence to COVID-19 SOPs (87.5%; 95% CI = 64.6–100) than food and beverages (40%; 95% CI = 15.2–64.8), plastics (60%; 95% CI = 17.1–100), as well as other types of industries (81.8%; 95% CI = 59–100). The relationship of overall adherence to SOPs with type of factories was however not significant (*X*^2^ = 7.04, *P* value = 0.071) (Table 4).

## 4. Discussion

Coronavirus disease (COVID-19) is an infectious disease caused by the SARS-CoV-2 virus and it was declared a pandemic by WHO in 2019 [1, 17]. No studies have been conducted in the factory settings in Uganda and hardly in the region, yet factory settings involve a high tendency of crowding which can increase the risk of exposure to COVID-19 [18].

No pharmaceutical interventions including personnel and public protective practices have been reported to play a significant role of preventing occurrence and spread of public health challenges, thereby promoting good livelihoods and minimizing the burden on the already strained healthcare systems [8, 19–21]. This study assessed the level of adherence to a number of NPIs set by the government of Uganda as SOPs to be implemented by factories as a prerogative for their opening and continued operation during the COVID-19 pandemic outbreak [4].

The level of adherence to the COVID-19 SOPs in the surveyed factories was noted to be higher compared to previous studies that investigated implementation of NPIs in public spaces [22–25]. In a 2021 study of selected super markets in Mukono and Kampala districts, only 16.6% complied with the COVID-19 guidelines [11], while in a countrywide survey conducted during the first outbreak phase in Uganda in 2020, a 29.0% level of compliance to SOPs was reported [9]. This study provided evidence of a significant improvement in the implementation of NPIs against COVID-19 which could probably be attributed to increased awareness of the public health implications of the outbreak that induced high vigilance among people with time [21, 26, 27]. The considerably high adherence to the SOPs could have resulted from active enforcement by company owners to avoid their operations being blocked by the government due to breach of Ministry of Health set guidelines for control of COVID-19 in factories [4].

In this study it was noted that communication and training of employees and wearing of personal protective equipment (PPE) and respiratory protection were the most implemented SOPs at the factories, followed by enhanced cleaning and disinfection of surfaces, exposure, and case reporting. Similar findings were obtained in a study conducted in supermarkets where 59.8% of the supermarkets regularly disinfected commonly touched surfaces, 44.5% provided their staff with job-specific training on infection prevention and control for COVID-19, and 54.4% of staff correctly used the PPE [10, 11]. On the contrary, some studies reported use of PPEs as the least observed SOP [9]. A number of studies revealed that implementation of a combination of some or all of these NPIs played a tremendous role of slowing down the COVID-19 outbreak and helped flatten its epidemic curve [24, 28, 29]. High compliance to implementation of the NPIs was however dependent on cost implications, whereby feasibly affordable interventions such as mask wearing, hand washing, and physical distancing were more emphasized [19, 20, 26, 30]. Compliance to set SOPs was also observed to increase with people's increasing beliefs regarding perceived effectiveness and convenience of use of particular interventions at their disposal [8, 27, 31].

This study showed that factories poorly adhered to ventilation, social distancing, health control plans, and display of COVID-19-related signage. Studies in Kampala and Ethiopia recorded much lower observance of social distancing (7.0% and 27.0%, respectively) [10, 32] as compared to this study. However, there are other studies that reported higher observance of social distancing (63% and 90%, respectively) [9, 12]. Ventilation is key in reducing the concentration of COVID-19 droplets released in the air of the factory settings, while social distancing limits the likelihood of spread [7, 8] Presence of signages on the factory premises and health control plans help in raising awareness as well enable swift and effective decision-making in COVID-19 mitigation [11, 33, 34].

Although good overall adherence to SOPs envisaged here was not dependent on location and type of factory, the study revealed that adherence to some SOPs such as exposure control plan, presence of signages on COVID-19, maintenance of social distance, and implementation of a health control plan varied with district of location. Likewise, exposure control plan, signages on COVID-19, and maintenance of social distance varied significantly with the type of factory. This could have probably been because follow-up and monitoring of this MOH directive on factories adherence to set SOPs was differently done by the task force on COVID-19 surveillance at districts. Variations noted for different types of factories could be attributed to varying space requirements and number of workers that needed to be streamlined before enforcement of these SOPs [33–36].

## 5. Conclusion

This study revealed moderately good overall adherence to COVID-19 SOPs by factories; however, variations in the level of implementation of individual SOPs were observed. The study recommends regular spot supervision of the factories by health authorities to strengthen enforcement of these guidelines during future pandemic outbreaks.

## Figures and Tables

**Table 1:** List of factories.

Location	Number	Factory	Factory type
Buikwe	1	Sezibwa Sugar Mill	Sugar manufacturing
	2	Kampala Salt Uganda Ltd	Salt production
	3	Yogi Steel Ltd	Steel manufacturing
	4	Modern Distillers Ltd	Beverage
	5	Gold Star Battery Uganda Limited	Solar battery manufacturing
	6	Bavima Steel Ltd	Steel manufacturing
	7	Leeko Uganda Limited	Soap, toothpaste, and Vaseline manufacturing
	8	Kayisylivan Nutritional Services	Beverage
	9	Kasaku Tea Estate	Tea factory
	10	Tembo steel Factory	Steel manufacturing

Wakiso	11	Cementers	Construction materials
	12	MEC Plastics	Plastics
	13	Parambot Distilleries	Beverage
	14	Excel Industries	Plastics
	15	Rohobuild	Construction materials
	16	Agri-exim Industries	Food processing
	17	Ishaka Quality Commodities Limited	Food processing
	18	Atis Biscuits (Baraji East Africa)	Food processing
	19	Chief Distilleries	Beverage
	20	Kiri Bottling Company	Food processing

Jinja	21	Ntake Bakery co. Limited	Food processing
	22	Najeru Packaging Industries limited	Fabrics/nonplastic packaging
	23	Makepasi Match limited	Wax matches
	24	Keshwala Industries Group	Beverage
	25	KABANDA and SANYU Grain Millers Group	Milling
	26	HEEK International Limited	Construction materials
	27	Akshar Agro Industries Limited	Agriengineering
	28	Elizabeth Queen Shoe Investment Limited	Foot wearing/shoes
	29	GEBAL Mineral Limited	Mining
	30	Nile Plywood Industries Limited	Timber products

Mukono	31	Good Brotherhood ICD	Ware house and shipping company
	32	Huaye	Goods transportation company
	33	Leaf tobacco and other commodities	Secondary production of cigarettes
	34	Hermian International Limited	Packaging of fruits (mangoes/blackjack)
	35	Huasheng International Limited	Production of plumbing materials
	36	Yuti Breweries	Beverage
	37	Marriat Limited	Plastic recycling company
	38	Medisell Limited	Medical and laboratory materials
	39	Mukwano Group Limited	Plastics production

**Table 2:** Demographics of the factories assessed.

District location	Type of factory				Kruskal–Wallis *X*^2^	*P* value
	Food and beverages, *n* (%)	Construction, *n* (%)	Plastics, *n* (%)	Others, *n* (%)		
Buikwe	4 (40.0)	4 (40.0)	0 (0.0)	2 (20.0)	0.82	0.08
Jinja	3 (30.0)	2 (20.0)	0 (0.0)	5 (50.0)		
Mukono	2 (22.2)	0 (0.0)	3 (33.3)	4 (44.4)		
Wakiso	6 (60.0)	2 (20.0)	2 (20.0)	0 (0.0)		
Total	15 (38.5)	8 (20.5)	5 (12.8)	11 (28.2)		

**Table 3:** Cross-tabulation of adherence to COVID-19 SOP attributes in factories with district of location.

Variable	Category	Buikwe *N* (%)	Jinja *N* (%)	Mukono *N* (%)	Wakiso *N* (%)	Kruskal–Wallis *X*^2^	*P* value
Exposure control plan	No	4 (40.0)	3 (30.0)	2 (22.2)	8 (80.0)	7.66	**0.05**
	Yes	6 (60.0)	7 (70.0)	7 (77.8)	2 (20.0)		

Ventilation	No	5 (50.0)	6 (60.0)	8 (88.9)	6 (60.0)	3.33	0.34
	Yes	5 (50.0)	4 (40.0)	1 (11.1)	4 (40.0)		

Communication and training of employees	No	2 (20.0)	4 (40.0)	0 (0.0)	2 (20.0)	4.53	0.21
	Yes	8 (80.0)	6 (60.0)	9 (100)	8 (80.0)		

Signage placed at workplaces	No	3 (30.0)	4 (40.0)	8 (88.9)	7 (70.0)	8.32	**0.04**
	Yes	7 (70.0)	6 (60.0)	1 (11.1)	3 (30.0)		

Maintenance of social distancing at factories	No	10 (100)	2 (20.0)	3 (33.3)	10 (100)	22.76	**<0.001**
	Yes	0 (0.0)	8 (80.0)	6 (66.7)	0 (0.0)		

Factory work practices	No	6 (60.0)	5 (50.0)	3 (33.3)	5 (50.0)	1.34	0.72
	Yes	4 (40.0)	5 (50.0)	6 (66.7)	5 (50.0)		

Enhanced cleaning and disinfection	No	1 (10.0)	3 (30.0)	2 (22.2)	4 (40.0)	2.45	0.48
	Yes	9 (90.0)	7 (70.0)	7 (77.8)	6 (20.7)		

Personal protective equipment (PPE) and respiratory protection	No	2 (20.0)	4 (40.0)	0 (0.0)	2 (20.0)	4.53	0.21
	Yes	8 (80.0)	6 (60.0)	9 (100)	8 (80.0)		

Exposure and case reporting	No	3 (30.0)	5 (50.0)	1 (11.1)	7 (70.0)	7.44	0.06
	Yes	7 (70.0)	5 (50.0)	8 (88.9)	3 (30.0)		

Health control plan	No	6 (60.0)	9 (90.0)	0 (0.0)	9 (90.0)	20.71	**<0.001**
	Yes	4 (40.0)	1 (10.0)	9 (100)	1 (10.0)		

Overall adherence to SOPs	Poor	3 (30.0)	4 (40.0)	1 (11.1)	6 (60.0)	5.02	0.17
	Good	7 (70.0)	6 (60.0)	8 (88.9)	4 (40.0)		

P values in bold show significant differences of variables between districts.

**Table 4:** Cross tabulation of adherence to COVID-19 SOPs attributes with type of factory.

Variable	Category	Construction	Food and beverages	Plastics	Others	Kruskal–Wallis *X*^2^	*P* value
Exposure control plan	No	3 (37.5)	10 (66.7)	1 (20.0)	3 (27.3)	5.55	0.14
	Yes	5 (62.5)	5 (33.3)	4 (80.0)	8 (72.7)		

Ventilation	No	4 (50.5)	10 (66.7)	4 (80.0)	7 (63.6)	1.25	0.74
	Yes	4 (50.5)	5 (33.3)	1 (20.0)	4 (36.4)		

Communication and training of employees	No	1 (12.5)	5 (33.3)	0 (0.0)	2 (18.2)	3.07	0.38
	Yes	7 (87.5)	10 (66.7)	5 (100)	9 (81.8)		

Signage	No	3 (37.5)	10 (66.7)	4 (80.0)	5 (45.4)	3.38	0.34
	Yes	5 (62.5)	5 (33.3)	1 (20.0)	6 (54.6)		

Social distance	No	6 (75.0)	12 (80.0)	4 (80.0)	3 (27.3)	8.86	**0.03**
	Yes	2 (25.0)	3 (20.0)	1 (20.0)	8 (72.7)		

Factory work practices	No	3 (37.5)	10 (66.7)	3 (60.0)	3 (27.3)	4.50	0.21
	Yes	5 (37.5)	5 (33.3)	2 (40.0)	8 (72.7)		

Enhanced cleaning and disinfection	No	2 (25.0)	5 (33.3)	2 (40.0)	1 (9.1)	2.52	0.47
	Yes	6 (75.0)	10 (63.7)	3 (60.0)	10 (90.9)		

Personal protective equipment (PPE) and respiratory protection	No	1 (12.5)	5 (33.3)	0 (0.0)	2 (18.2)	3.07	0.38
	Yes	7 (87.5)	10 (66.7)	5 (100)	9 (81.8)		

Exposure and case reporting	No	1 (12.5)	11 (73.3)	2 (40.0)	2 (18.2)	11.24	**0.01**
	Yes	7 (87.5)	4 (27.7)	3 (60.0)	9 (81.8)		

Health control	No	5 (62.5)	11 (73.3)	2 (40.0)	6 (54.6)	2.04	0.56
	Yes	3 (37.5)	4 (26.7)	3 (60.0)	5 (45.4)		

Overall adherence	No	1 (12.5)	9 (60.0)	2 (40.0)	2 (18.2)	7.04	0.07
	Yes	7 (87.5)	6 (40.0)	3 (60.0)	9 (81.8)		

P values in bold show variables noted to vary significantly among the types of factories.

## Data Availability

All data supporting the results of this study have been submitted as a supplementary file.

## Abbreviations

WHO: World Health Organization
COVID-19: Coronavirus disease
SOPs: Standard operating procedures
MoH: Ministry of Health
PPE: Personal protective equipment
AFROHUN: Africa One Health University Network
95% CI: 95% confidence interval.

## Supplementary Materials

Supplementary Materials Demographic characteristics of study factories and their adherence to COVID-19 Standard Operating Procedures attributes.

## References

[1] WHO (2021). Coronavirus (COVID- 19) Dashboard with Vaccination Data. https://covid19.who.int/.

[2] Republic of Uganda M of H (2020). Uganda confirms 1st case of covid-19. *Electronic Journal of Medical and Educational Technologies*.

[3] Anguzu R., Kabagenyi A., Cassidy L. D. (2022). Adherence to COVID-19 preventive measures and its association with intimate partner violence among women in informal settings of Kampala, Uganda. *PLOS Global Public Health*.

[4] The Republic of Uganda (2020). *The republic of uganda Progress in Implementing the Standard Operating Procedures by the Trade Industry and Cooperatives Sector during the Coronavirus/Covid-19*.

[5] Oppong R (2020). Looking beyond the impact of covid-19: the economist view. *Financ Manag Engineering Journal Africa*.

[6] Bo Y., Guo C., Lin C. (2021). Effectiveness of non-pharmaceutical interventions on COVID-19 transmission in 190 countries from 23 January to 13 April 2020. *International Journal of Infectious Diseases*.

[7] Heidary S. M., Sorouri S., Naseri M., Malakoti N., Movahedinia S., Shakeri F. (2023). An overview of existing evidence for non-pharmacological interventions against COVID-19 transmission. *Med Edu Bull*.

[8] Zhang W., Wu Y., Wen B. (2023). Non-pharmaceutical interventions for COVID-19 reduced the incidence of infectious diseases: a controlled interrupted time-series study. *Infect Dis Poverty*.

[9] Amodan B. O., Bulage L., Katana E. (2020). Level and determinants of adherence to COVID-19 preventive measures in the first stage of the outbreak in Uganda. *International Journal of Environmental Research and Public Health*.

[10] Bante A., Mersha A., Tesfaye A. (2021). Adherence with COVID-19 preventive measures and associated factors among residents of Dirashe district, southern Ethiopia. *Patient Preference and Adherence*.

[11] Mugambe R. K., Ssekamatte T., Kisaka S. (2021). Extent of compliance with COVID-19 prevention and control guidelines among supermarkets in Kampala Capital City and Mukono Municipality, Uganda. *PLoS One*.

[12] Kudamba A., Walusansa A., Ssenku J. E. (2021). Assessment of the adherence to standard operating Procedures of covid-19 among market vendors in sironko district. *Asian Journal of Medicine and Health*.

[13] Kebede Y., Yitayih Y., Birhanu Z., Mekonen S., Ambelu A. (2020). Knowledge, perceptions and preventive practices towards COVID-19 early in the outbreak among Jimma university medical center visitors, Southwest Ethiopia. *PLoS One*.

[14] Oriesek D. F. (2020). Non-pharmaceutical interventions in the fight against pandemics now and then. *A Comparison Between the Current Covid-19 Pandemic and the 1918/19 Influenza Pandemic Commonly Known as the Spanish Flu*.

[15] Panthy L., Panthi J., Amgain K., Thapaliya P., Laar J. V. (2020). COVID-19 in Nepal: scarcity of personal protective equipment (PPE) and its alternative. *Europasian Journal of Medical Sciences*.

[16] National Institute of Environmental Health Sciences (2023). Key elements of a model workplace safety and health covid-19 vaccination program. https://tools.niehs.nih.gov.

[17] Bell D., Hansen K. S., Kiragga A. N., Kambugu A., Kissa J., Mbonye A. K. (2020). Predicting the impact of COVID-19 and the potential impact of the public health response on disease burden in Uganda. *The American Journal of Tropical Medicine and Hygiene*.

[18] Caduff C. (2020). What went wrong: corona and the world after the full stop. *Medical Anthropology Quarterly*.

[19] Kantor B. N., Kantor J. (2020). Non-pharmaceutical interventions for pandemic COVID-19: a cross-sectional investigation of US general public beliefs, attitudes, and actions. *Frontiers of Medicine*.

[20] Solomon H., Thea D. M., Galea S., Sabin L. L., Lucey D. R., Hamer D. H. (2022). Adherence to and enforcement of non-pharmaceutical interventions (NPIs) for COVID-19 prevention in Nigeria, Rwanda, and Zambia: a mixed-methods analysis. *PLOS Global Public Health*.

[21] Fitzpatrick A., Beg S., Derksen L. (2021). Health knowledge and non-pharmaceutical interventions during the Covid-19 pandemic in Africa. *Journal of Economic Behavior & Organization*.

[22] Usman I. M., Ssempijja F., Ssebuufu R. (2020). Community drivers affecting adherence to WHO guidelines against COVID-19 amongst rural Ugandan market vendors. *Frontiers in Public Health*.

[23] Kasozi K. I., MacLeod E., Ssempijja F. (2020). Misconceptions on COVID-19 risk among Ugandan men: results from a rapid exploratory survey, April 2020. *Frontiers in Public Health*.

[24] Ssebuufu R., Sikakulya F. K., Mambo S. B. (2020). Knowledge, attitude, and self-reported practice toward measures for prevention of the spread of COVID-19 among Ugandans: a nationwide online cross-sectional survey. *Frontiers in Public Health*.

[25] Methodius T., Musewa A., Mirembe B. B. (2023). Knowledge, attitudes, and adherence relating to COVID-19 and its prevention measures in high-risk districts of Uganda in 2020. *Frontiers in Epidemiology*.

[26] Owhonda G. (2021). Community awareness, perceptions, enablers and potential barriers to non-pharmaceutical interventions (NPIs) in the COVID-19 pandemic in rivers state, Nigeria. *Biomedical Journal of Scientific & Technical Research*.

[27] Barbeito I., Precioso D., Sierra M. J. (2023). Effectiveness of non-pharmaceutical interventions in nine fields of activity to decrease SARS-CoV-2 transmission (Spain, September 2020–May 2021). *Frontiers in Public Health*.

[28] Worby C. J., Chang H. H. (2020). Face mask use in the general population and optimal resource allocation during the COVID-19 pandemic. *Nature Communications*.

[29] Li T., Liu Y., Li M., Qian X., Dai S. Y. (2020). Mask or no mask for COVID-19: a public health and market study. *PLoS One*.

[30] Resolve To Save Lives (2020). Implementation of non-pharmaceutical interventions. evidence base and application to the african context. *Init vital strategies*.

[31] Rwagasore E., Nsekuye O., Rutagengwa A., El-Khatib Z. (2023). Effect of non-pharmaceutical interventions on COVID-19 in Rwanda: an observational study. *J Epidemiol Glob Health*.

[32] UNICEF (2022). *Uganda Country Office*.

[33] Davey S. (2020). Impact of social distancing on curtailing COVID 2019 epidemic in India: a systematic review by swot analysis approach. *Epidemiology International*.

[34] Travica B. (2020). Containment strategies for COVID-19 pandemic. *SSRN Electronic Journal*.

[35] Siedner M. J., Harling G., Reynolds Z. (2020). Social distancing to slow the US COVID-19 epidemic: longitudinal pretest–posttest comparison group study. *PLoS Medicine*.

[36] Teslya A, Pham T. M., Godijk N. G., Kretzschmar M. E., Bootsma M. C. J., Rozhnova G. (2020). Impact of self-imposed prevention measures and short-term government-imposed social distancing on mitigating and delaying a COVID-19 epidemic: A modelling study. *PLoS Med*.

